# Neck and Back Sprain and Hand Flexor Tendon Repair Are More Common in Victims of Domestic Violence Compared With Patients Who Were Not Victims of Domestic Violence: A Comparative Study of 1,204,596 Patients Using the National Trauma Data Bank

**DOI:** 10.5435/JAAOSGlobal-D-21-00124

**Published:** 2021-09-02

**Authors:** Teresa C. Logue, Nicholas C. Danford, Elise C. Bixby, Matthew M. Levitsky, Melvin P. Rosenwasser

**Affiliations:** From the Columbia University Vagelos College of Physicians and Surgeons (Logue), Department of Orthopaedic Surgery (Dr. Danford, Dr. Bixby, Dr. Levitsky, Dr. Rosenwasser), Columbia University Irving Medical Center, New York, NY.

## Abstract

The purpose of this study was to determine the most common orthopedic diagnoses and procedures among patients who experience domestic violence (DV) and to determine whether these were more common in patients who experienced DV compared with those who did not.

**Methods:**

We performed a retrospective cohort study of all patients identified in the National Trauma Data Bank. Patients were divided into two cohorts for comparison: victims of DV and all other patients. The main outcome measurements were a diagnosis of an orthopedic injury and/or a procedure performed for an orthopedic diagnosis.

**Results:**

In total, 1,204,596 patients were included in the analysis, of whom 3191 (0.26%) were victims of DV. Adult trauma patients with DV were more likely to have a diagnosis of neck and back sprain (odds ratio 1.98, 95% confidence interval 1.60 to 2.44, *P* < 0.0001) and more likely to undergo surgical repair of the flexor tendon of the hand (odds ratio 2.76, 95% confidence interval 1.75 to 4.35, *P* < 0.0001) than patients without a diagnosis of DV.

**Discussion:**

Patients who experience DV were more likely to have back and neck sprain and more likely to undergo repair of flexor tendon of the hand than those who do not experience DV.

Domestic violence (DV) is a common problem that is associated with immediate and long-term health consequences. Patients who experience DV frequently present to orthopedic surgeons, who are well positioned to identify victims and refer them to support services.^1,2,3,4,5^ Effective intervention can reduce future exposure to abuse.^6^

Craniofacial injuries and multiple fractures at various stages of healing are associated with DV in adults, yet few studies have described specific orthopedic diagnoses and orthopedic procedures that patients undergo in the setting of DV.^2,3,7,8,9,10,11,12,13,14,15^ Data we do have suggest that back (including neck) and hand injuries are the most common orthopedic diagnoses among patients who experience DV. Upper extremity injuries may be more common in this population compared with injuries of the lower extremity.^7,9,14,16^ Importantly, these data are commonly derived from an outpatient setting, which may limit its applicability to a population presenting emergently to trauma centers. Moreover, we do not know whether these diagnoses are more common in patients who are victims of DV compared with those who are not.^5,7^

The purpose of this study was to determine the most common orthopedic diagnoses and procedures among patients who experience DV and to determine whether these injuries and procedures were more common in patients who experienced DV compared with those who do not. Better characterization of injuries and procedures will help orthopedic surgeons identify and screen for domestic abuse when a patient presents emergently after trauma.

We hypothesized that the most common orthopedic diagnoses among adults with DV would be back (including neck) fracture or sprain followed by a hand injury (fracture or tendon injury) because these diagnoses were among those most frequently reported in the literature.^7,9,16^ We also hypothesized that procedures performed on the hand would be most common in this population, given the frequency of hand injuries reported in the literature.^7,9,16^ Finally, we hypothesized that these diagnoses and procedures would occur in a markedly greater percentage of DV patients compared with patients who did not experience DV.

## Methods

### Study Design, Participants, and Data Collection

We conducted a retrospective analysis of the National Trauma Data Bank (NTDB) data from the years 2011 to 2013. The NTDB is a prospectively collected registry of trauma data maintained by the Committee on Trauma of the American College of Surgeons. With more than 7.5 million patient records from more than 900 trauma centers, it is the largest repository of trauma data in the world.^17^ As a convenience sample, the NTDB is not nationally representative of all trauma incidents in the United States, although it is considered to be representative of all level I/II trauma facilities.^17^ The data set is deidentified, and no protected health information is provided.

Inclusion criteria were patients in the NTDB presenting from 2011 to 2013 aged 18 years or older. Patients were divided into two groups for comparison. The first group consisted of patients who did not have a diagnosis associated with DV based on *International Classification of Diseases, Ninth Revision, Clinical Modification* (*ICD-9 CM*) coding. The second group consisted of patients identified by *ICD-9 CM* diagnosis codes 995.80 to 995.85 and/or an *ICD-9 CM* etiology code of E967.0 to E967.9 (Supplemental Table 1, http://links.lww.com/JG9/A153). This diagnosis grouping allowed us to collect data on experience of DV perpetrated by a spouse, partner, ex-spouse, or ex-partner (intimate partner) and domestic abuse by nonpartner perpetrators including parents, step-parents, children, siblings, grandparents, other relatives, nonrelated caregivers, or unspecified persons (Supplemental Table 1, http://links.lww.com/JG9/A153). This group was divided into two subgroups for additional analysis. One subgroup of patients had a diagnosis of abuse by an intimate partner (*ICD-9 CM* etiology code E967.3), and a second subgroup of patients had a diagnosis of abuse by a nonintimate relation (child, parents, sibling, grandparent, other relative, and nonrelated caregiver) (Supplemental Table 1, http://links.lww.com/JG9/A153). The NTDB derives these diagnoses from a pre-established data source hierarchy that includes hospital discharge summary, billing sheet/medical records coding summary sheet, trauma flow sheet, and emergency department and intensive care unit records. Exclusion criteria were patients younger than 18 years of age and patients with missing demographic data.

### Variables

Demographic data were age, sex, race, comorbidities (alcoholism, current smoker, diabetes mellitus types I or II, functionally dependent health status, obesity, and cirrhosis), payment method, and hospital teaching status. Injury characteristics, hospital course, and outcome data were injury type (blunt, burn, penetrating, and other), injury intent (assault, self-inflicted, unintentional, and undetermined), injury mechanism (cut/pierce, fall, burn, firearm, struck, suffocation, and other), injury severity score (ISS), location at time of injury, alcohol use status, and mortality. Injury type, intent, and mechanism were populated based on the primary etiology code associated with the trauma incident only. For incidents with a primary etiology code in E967.0 to E967.9, which contains the identity of the perpetrator of adult abuse but not the nature of injury, the assigned injury type and mechanism were “other/unspecified.”

The combined primary outcome variables were the most common orthopedic diagnoses and most common orthopedic procedures identified through *ICD-9 CM* diagnosis and procedure codes. We defined orthopedic diagnosis and orthopedic procedure as any injury associated with the spine, pelvis, or appendicular skeleton. For the purposes of our study, we did not consider head, face, chest including ribs, or isolated skin (integumentary system) injuries to be orthopedic diagnoses nor did we consider procedures on these regions orthopedic procedures. This definition was determined a priori because our study purpose was to define orthopedic diagnoses and procedures that would assist the orthopedic surgeon who is consulted specifically for musculoskeletal injuries to assess for DV. Associated injuries to the head, face, chest, and skin are well-described elsewhere.^7,8,9,10,11,12,13,14^

### Statistical Analyses

Descriptive statistics were percent of total study population for categorical variables. The DV and non-DV groups were compared across categorical demographic variables using Pearson chi-squared analysis. Bivariate analysis was then done to compare the rates of DV-associated orthopedic diagnoses and procedures between groups. Notable results from the bivariate analyses were used in an adjusted multivariate regression to compare the prevalence of these diagnoses and procedures in the DV group with that of the nonviolence group. Multivariate analysis was used preferentially over propensity score matching because of the high number of events per confounding variable.^18^ To correct for multiple group comparisons, a Bonferroni correction was applied with statistical significance set to *P* < 0.05 at baseline. All statistical analyses were conducted using SAS 9.4 (SAS Institute).

## Results

A total of 1,983,764 patients were identified, with 779,168 excluded because of missing demographic or outcome data. We therefore analyzed the records of 1,204,596 patients. Of these, 3,191 (0.26%) had an *ICD-9 CM* code consistent with DV and 1,201,405 (99.7%) did not. In total, 1168 (36.6%) of those with reported DV had a perpetrator who was a spouse, partner, ex-spouse, or ex-partner (Supplemental Table 1, http://links.lww.com/JG9/A153).

Report of DV varied markedly by age; sex; race; selected comorbidities; payment method; hospital teaching status; injury type, intent, mechanism, and severity score (ISS); and location, alcohol use status, and whether death occurred. Trauma patients with a diagnosis of DV were more likely to be young (aged 18 to 33 years; *P* < 0.0001), female (*P* < 0.0001), Black, Native American or White, and non-Hispanic (all *P* < 0.0001); have alcoholism (*P* < 0.0001); smoke (*P* < 0.0001); pay for care with Medicaid or self-pay (both *P* < 0.0001); and seek care at community and university hospitals (*P* < 0.0001) than those without a diagnosis of DV. DV patients were also more likely to have used alcohol (*P* < 0.0001) and have less severe injuries (ISS less than 15, *P* < 0.0001). Their injuries were more likely to have occurred at home (*P* < 0.0001); from intentional assault (*P* < 0.0001); with mechanisms of cutting/piercing, firearms, and being struck (*P* < 0.0001); and result in penetrating trauma (*P* < 0.0001). Adult trauma patients with DV were less likely to die during hospitalization than those without (*P* < 0.0001) (Supplemental Table 2, http://links.lww.com/JG9/A154).

The most common orthopedic diagnosis was vertebral fracture (ICD code 805.2 and 805.4, closed fracture of thoracic or lumbar vertebra without mention of spinal cord injury). Although the patients with a diagnosis of DV were not more likely to sustain a vertebral fracture compared with patients who did not experience DV, younger patients aged 18 to 33 years with a diagnosis of vertebral fracture were more likely to experience DV compared with older patients aged 70 to 89 years presenting with the same diagnosis (*P* = 0.006) (Table 1 and Supplemental Table 4, http://links.lww.com/JG9/A156).

**Table 1:** Odds Ratios of Orthopedic Diagnoses by Experience of Domestic Violence

Diagnosis	n	Experienced Domestic Violence		Multivariate Analysis			*P* value^a^
		Yes (% DV Patients)	No (% non-DV Patients)	OR	95% CI		
Vertebral fracture	136,623	3.4	11.4	1.21	0.99	1.48	1.00
Neck/back sprain	34,606	3.2	2.9	1.98	1.60	2.44	**<0.0001**
Ankle fracture	41,849	1.0	3.5	0.78	0.55	1.11	1.00
Hand fracture/tendon injury	19,296	1.7	1.6	1.38	1.05	1.82	0.18
Radius/ulna fracture	14,433	0.6	1.2	1.13	0.70	1.83	1.00
Tibia/fibula fracture	63,808	1.6	5.3	1.20	0.91	1.59	1.00
Digital nerve injury	2548	0.6	0.2	2.59	1.61	4.17	**0.0007**
Humerus fracture	9,919	0.3	0.8	1.39	0.75	2.58	1.00

CI = confidence interval, DV = domestic violence

a *P* value after Bonferroni correction for multiple comparisons. Significant values are defined as <0.05 and are presented in bold.

In the adjusted multivariate analysis, adult trauma patients with DV were more likely to have orthopedic diagnoses of neck and back sprain (odds ratio [OR] 1.98, 95% confidence interval [CI] 1.60 to 2.45, adjusted *P* < 0.0001) and digital nerve injury (OR 2.59, 95% CI 1.61 to 4.17, adjusted *P* = 0.0007) compared with cohort of patients who did not experience DV (Table 1). For orthopedic procedures, DV patients were more likely to undergo surgical repair of the flexor tendon of the hand (OR 2.76, 95% CI 1.75 to 4.35, adjusted *P* < 0.0001) and less likely to undergo open reduction and internal fixation of the femur (OR 0.484, 95% CI 0.32 to 0.73, adjusted *P* = 0.0052) than those without DV (Table 2).

**Table 2:** Odds Ratios of Undergoing Orthopedic Procedures by Experience of Domestic Violence

Procedure	n	Experienced Domestic Violence		Multivariate Analysis			*P* value^a^
		Yes (% DV patients)	No (% non-DV patients)	OR	95% CI		
Any procedure	1,075,336	87.1	89.3	0.91	0.82	1.02	0.71
Procedure							
Reduction with internal fixation or application of fixator device, tibia/fibula	107,297	2.5	8.9	0.88	0.70	1.11	1.00
Open reduction with internal fixation, radius/ulna	42,703	1.4	3.6	1.04	0.76	1.41	1.00
Open reduction with internal fixation, humerus	22,230	0.9	1.9	1.04	0.72	1.52	1.00
Open reduction with internal fixation, femur	63,088	0.7	5.3	0.48	0.32	0.73	0.005
Closed reduction with internal fixation, femur	35,271	0.7	2.9	0.95	0.61	1.47	1.00
Surgical repair of flexor tendon of the hand	2957	0.7	0.2	2.76	1.75	4.35	**<0.0001**
Open reduction with internal fixation, carpals, metacarpals, and/or phalanges	11,913	0.5	1.0	0.98	0.60	1.59	1.00

CI = confidence interval, DV = domestic violence

a *P* value after Bonferroni correction for multiple comparisons. Significant values are defined as < 0.05 and are presented in bold.

Our subgroup analysis of adult trauma patients with a diagnosis of DV perpetrated by an intimate partner compared with those with a nonpartner perpetrator identified DV victims perpetrated by a partner as more likely to be female (*P* < 0.0001), Black (*P* < 0.0001), pay for care through Medicaid (*P* = 0.002), and seek care at a university hospital (*P* < 0.0001) and less likely to have used alcohol beyond the legal limit (*P* = 0.0037) (Supplemental Table 3, http://links.lww.com/JG9/A155). Patients with intimate partner violence were less likely to have a procedure than those with a nonpartner perpetrator in bivariate analysis (*P* = 0.009), although this difference was not significant in multivariate analysis. Overall, the intimate partner and nonpartner subgroups did not have significantly different patterns of orthopedic diagnoses or procedures (Tables 3 and 4).

**Table 3:** Odds Ratios of Having Orthopedic Diagnoses by Whether the Abuse Was Perpetrated by an Intimate Partner

Diagnosis	n	Abuse by Partner?		Multivariate Analysis			*P* value^a^
		Yes (% DV patients)	No (% DV patients)	OR	95% CI		
Vertebral fracture	109	4.1	3.0	1.26	0.84	1.91	1.00
Neck/back sprain	102	3.7	2.9	0.93	0.61	1.43	1.00
Ankle fracture	33	1.2	0.9	1.05	0.50	2.20	1.00
Hand fracture/tendon injury	54	2.2	1.4	1.37	0.77	2.46	1.00
Radius/ulna fracture	18	0.6	0.5	1.07	0.38	3.01	1.00
Tibia/fibula fracture	52	1.8	1.5	1.11	0.60	2.05	1.00
Digital nerve injury	19	0.8	0.5	1.31	0.50	3.40	1.00
Humerus fracture	11	0.3	0.4	0.77	0.21	2.86	1.00
Any procedure	2778	84.5	88.5	0.90	0.72	1.14	1.00

CI = confidence interval, DV = domestic violence

a *P* value after Bonferroni correction for multiple comparisons.

**Table 4:** Odds Ratios of Undergoing Orthopedic Procedures by Whether Abuse Was Perpetrated by an Intimate Partner

Procedure	n	Abuse by Partner		Multivariate Analysis			*P* value^a^
		Yes (% DV patients	No (% DV patients)	OR	95% CI		
Any procedure	2,778	84.5	88.5	0.90	0.72	1.14	1.00
Procedure							
Reduction with internal fixation or application of fixator device, tibia/fibula	79	2.9	2.2	1.14	0.69	1.89	1.00
Open reduction with internal fixation, radius/ulna	43	1.5	1.2	1.16	0.60	2.24	1.00
Open reduction with internal fixation, humerus	29	1.1	0.8	1.14	0.52	2.54	1.00
Open reduction with internal fixation, femur	23	0.4	0.9	0.58	0.19	1.74	1.00
Closed reduction with internal fixation, femur	21	0.6	0.7	1.02	0.38	2.72	1.00
Surgical repair of flexor tendon of the hand	21	1.0	0.4	2.48	0.97	6.37	0.47
Open reduction with internal fixation, carpals, metacarpals, and/or phalanges	17	0.3	0.6	0.61	0.18	2.00	1.00

CI = confidence interval, DV = domestic violence

a *P* value after Bonferroni correction for multiple comparisons.

## Discussion

Our hypothesis that the most common orthopedic diagnoses among adults who experienced DV would be back and neck injuries followed by injuries involving the hand was validated. Vertebral fracture and neck/back sprain were the two most common diagnoses (combined 6.6% of DV patients), followed by hand injury (fracture or tendon injury, 1.7%), which was followed by ankle fracture (1.0%), and radius or ulnar fracture (0.6%). Our hypothesis that procedures on the hand would be most common in this population was not validated because fixation of tibial or fibula fractures was most common (2.5% of abuse patients). Our hypothesis that these orthopedic diagnoses would be present in a significantly greater percentage of patients with DV than patients who did not experience domestic abuse was validated regarding only neck and back sprain (3.2% versus 2.9%, *P* < 0.0001). Finally, our hypothesis that patients with a diagnosis of DV would be more likely to undergo procedures of the hand was partially validated because patients with DV were more likely to undergo repair of flexor tendon of the hand compared with the control group (*P* < 0.0001).

Our study is an important addition to the literature of DV and its intersection with orthopedic surgery. Our finding that the diagnoses of back and/or neck sprain and hand injuries are among the common orthopedic diagnoses in patients who experience DV agrees with previous literature.^7,14,16^ Importantly, we add to this literature by identifying patients with DV diagnoses and undergoing procedures for these injuries in a population of patients presenting emergently to trauma centers. Other data of DV victims and associated orthopedic injuries are mostly from the outpatient setting.^5,7^ With these data from the NTDB, clinicians have to rely less on extrapolation for identifying potential victims of abuse in the emergency department.

We identified higher rates of DV among women and among Black, Hispanic, and Native American patients, which aligns with previously published data.^19,20^ Our finding that a markedly higher proportion of DV patients sustained neck and/or back sprain and digital nerve injury compared with the rest of the study population is a novel finding which should be investigated further because it may relate to mechanism of injury, such as a knife laceration injury. We did not find a notable increase in forearm fractures among patients with DV compared with nondomestic patients, which suggests that self-defense or “nightstick” injuries may not help identify victims of abuse, as has been suggested.^21,22^

DV is commonly associated with young women. The United States Preventive Services Task Force recommends universal screening for intimate partner violence in women of reproductive age.^6^ However, abuse affects a broad range of people. We found that 27.0% of patients with DV were older than 49 years, and 37.5% were men. We therefore emphasize the need to retain vigilance for the diagnosis of DV among all segments of the orthopedic population. The American Academy of Orthopaedic Surgeons recommends that orthopedic surgeons develop skills to screen patients with relevant injuries for DV and offer support to those who screen positive, yet patients with partner violence who present to orthopedic care are rarely asked about abuse history.^1,4,5^ Surveys of orthopedic trauma surgeons in the United States and Canada suggest that one barrier to victim identification may be provider discomfort with recognizing signs of DV.^23,24^ Routine screening for DV in orthopedic settings has been proposed,^2,3^ and our findings support universal screening for DV in orthopedic patients who present to emergency departments. However, until this is implemented, orthopedic surgeons must at the very least perform additional screening for DV in all adult trauma patients with suspect injuries or a documented history of DV. We believe this is an ethical responsibility of the orthopedic surgeon because the purpose of a physician is to identify and treat a problem that a patient presents with. If treatment is not possible or feasible, a pathway toward treatment (eg, in the form of a referral) should be initiated.

Strengths of our study are the large sample size, the comparison cohort, and the objective measure of diagnosis through *ICD-9 CM* coding. Limitations of our study include potential underreporting of DV. We identified 2.6 DV cases per 1000 adult trauma center discharges (0.26%) among our study population, a diagnosis rate that is similar to previous studies using administrative data sets,^25^ although far lower than what has been estimated based on patient questionnaires administered at outpatient fracture clinics.^4,5^ However, DV is considered underreported and under-documented in the setting of trauma.^26^ Even among the general population, just 12% to 17% of female patients who self-report physical violence have it documented in the medical record.^3,27^ Barriers to reporting and documentation of DV include physicians' lack of accurate diagnosis and coding error. Nonabuse diagnoses are often coded first possibly because treatment for DV is poorly reimbursed.^28-30^ We were unable to determine how DV codes were assigned by individual treating providers; we do not know whether the determination was based on results from a screening tool or voluntary self-report, for example. Possibly, the way in which DV was diagnosed varied by trauma center or even by the provider within individual trauma centers. Despite this limitation, we still were able to analyze a large cohort of patients who experienced DV. Another limitation is lack of certain data that may have affected our outcomes. Specifically, we did not analyze patient education level, income, and pregnancy status because this information was not reported in NTDB during the years 2011 to 2013. Finally, we are unable to further characterize the nature of the DV-associated injuries we report because of lack of specificity in the ICD-9 codes. For example, we are unable to determine whether vertebral fractures were treated operatively or nonoperatively, whether they were high-energy versus low-energy, or whether they were associated with neurologic deficits. We did find that younger patients with a diagnosis of vertebral fracture were more likely to experience DV compared with older patients with the same diagnosis, which suggests that treatment, energy level, and associated deficits may differ among different segments of the population we studied. Older patients with this diagnosis may have an osteoporotic insufficiency fracture, whereas a younger patient may present with a fracture from a higher energy mechanism of injury.

Additional research areas identified by our study include an analysis of variables associated with the higher rates of DV in patients who sought care at teaching hospitals versus nonteaching hospitals. Possibly, this difference reflects differences in patient populations, but the disparity might also reflect different screening or reporting practices between hospital settings.

In conclusion, vertebral fracture and neck/back sprain are the most common orthopedic diagnoses in patients who experienced DV and presented emergently to a trauma center in the United States. Patients who experience DV are more likely to have back and neck sprain and more likely to undergo repair of flexor tendon of the hand than those who do not experience domestic violence.

## Supplementary Material

SUPPLEMENTARY MATERIAL
