# Supplementary material for: Neck and Back Sprain and Hand Flexor Tendon Repair Are More Common in Victims of Domestic Violence Compared With Patients Who Were Not Victims of Domestic Violence: A Comparative Study of 1,204,596 Patients Using the National Trauma Data Bank
Source: J Am Acad Orthop Surg Glob Res Rev. 2021 Sep 2;5(9):e21.00124. doi: 10.5435/JAAOSGlobal-D-21-00124 (PMC8416016; doi:10.5435/JAAOSGlobal-D-21-00124)
Supplement: SUPPLEMENTARY MATERIAL [file jagrr-5-e21.00124-s001.docx]

Supplemental Table 1

| **Table 1: Included Patients** |  |  |
| --- | --- | --- |
| **ICD9 Etiology Code** | **ICD9 Code Description** | **n** |
| E967.0 | Perpetrator of adult abuse, by father, stepfather, or boyfriend | 656 |
| E967.1 | Perpetrator of adult abuse, by other specified person | 221 |
| E967.2 | Perpetrator of adult abuse, by mother, stepmother, or girlfriend | 157 |
| E967.3 | Perpetrator of adult abuse, by spouse or partner | 1168 |
| E967.4 | Perpetrator of adult abuse, by child | 272 |
| E967.5 | Perpetrator of adult abuse, by sibling | 135 |
| E967.6 | Perpetrator of adult abuse, by grandparent | 2 |
| E967.7 | Perpetrator of adult abuse, by other relative | 138 |
| E967.8 | Perpetrator of adult abuse, by non-related caregiver | 22 |
| E967.9 | Perpetrator of adult abuse, by unspecified person | 346 |
| **ICD9 Diagnosis Code** |  |  |
| 995.80 | Unspecified adult maltreatment | 4 |
| 995.81 | Adult physical abuse | 136 |
| 995.82 | Adult emotional/psychological abuse | 3 |
| 995.83 | Adult sexual abuse | 15 |
| 995.84 | Adult neglect (nutritional) | 3 |
| 995.85 | Other adult abuse and neglect | 2 |
|  |  |  |
| Total* |  | 3191 |
|  |  |  |
| *74 patients had a diagnosis code consistent with domestic violence only, 3028 had an etiology code consistent with domestic violence only, and 89 had both a diagnosis and etiology code consistent with domestic violence. | | |
| ICD = International Classification of Disease | |  |
