# Supplementary material for: Neck and Back Sprain and Hand Flexor Tendon Repair Are More Common in Victims of Domestic Violence Compared With Patients Who Were Not Victims of Domestic Violence: A Comparative Study of 1,204,596 Patients Using the National Trauma Data Bank
Source: J Am Acad Orthop Surg Glob Res Rev. 2021 Sep 2;5(9):e21.00124. doi: 10.5435/JAAOSGlobal-D-21-00124 (PMC8416016; doi:10.5435/JAAOSGlobal-D-21-00124)
Supplement: SUPPLEMENTARY MATERIAL [file jagrr-5-e21.00124-s002.docx]

Supplemental Table 2

| **Supplemental Table 2: Baseline Patient, Injury and Hospital Characteristics** | | |  |  |  |  |
| --- | --- | --- | --- | --- | --- | --- |
|  |  |  | **Experienced domestic violence** | |  |  |
|  |  |  | **Yes** | **No** | ***P-value (raw)*** | ***P-value (Bonferroni)*** |
|  | **n** | **Percent total** |  |  |  |  |
| **All patients** | 1,204,596 | 100% | 0.3% | 99.7% |  |  |
| n, Age 18-33 | 312,374 | 25.9% | 41.1% | 25.9% | <.0001 | <.0001 |
| n, Age 34-48 | 223,265 | 18.5% | 31.9% | 18.5% |  |  |
| n, Age 49-69 | 355,261 | 29.5% | 22.5% | 29.5% |  |  |
| n, Age 70-89 | 313,696 | 26.0% | 4.5% | 26.1% |  |  |
| Male | 733,747 | 60.9% | 37.5% | 61.0% | <.0001 | <.0001 |
| Female | 470,849 | 39.1% | 62.5% | 39.0% |  |  |
|  |  |  |  |  |  |  |
| **Race** |  |  |  |  |  |  |
| White, Non-Hispanic | 734,739 | 61.0% | 40.7% | 61.1% | <.0001 | <.0001 |
| Black | 142,441 | 11.8% | 30.4% | 11.8% |  |  |
| Native American | 9,309 | 0.8% | 3.4% | 0.8% |  |  |
| White, Hispanic | 24,271 | 2.0% | 4.2% | 2.0% |  |  |
| Other | 119,878 | 10.0% | 12.5% | 9.9% |  |  |
| Unknown* | 173,958 | 14.4% | 8.7% | 14.5% |  |  |
|  |  |  |  |  |  |  |
| **Comorbidities** |  |  |  |  |  |  |
| Alcoholism | 114,087 | 9.5% | 17.1% | 9.5% | <.0001 | <.0001 |
| Current smoker | 204,008 | 16.9% | 33.0% | 16.9% | <.0001 | <.0001 |
| Diabetes mellitus | 159,837 | 13.3% | 6.8% | 13.3% | <.0001 | <.0001 |
| Functionally dependent health status | 23,019 | 1.9% | 0.6% | 1.9% | <.0001 | <.0001 |
| Obesity | 69,612 | 5.8% | 6.0% | 5.8% | 0.62 | 1.0000 |
| Cirrhosis | 7,513 | 0.6% | 0.6% | 0.6% | 0.98 | 1.0000 |
|  |  |  |  |  |  |  |
| **Primary Payment Method** |  |  |  |  |  |  |
| Medicaid | 104,908 | 8.7% | 25.3% | 8.7% | <.0001 | <.0001 |
| Self Pay | 186,999 | 15.5% | 30.6% | 15.5% |  |  |
| Private/commercial insurance | 318,268 | 26.4% | 18.4% | 26.4% |  |  |
| Medicare | 350,414 | 29.1% | 12.7% | 29.1% |  |  |
| Other | 244,007 | 20.3% | 13.1% | 20.3% |  |  |
|  |  |  |  |  |  |  |
| **Hospital Teaching Status** |  |  |  |  |  |  |
| Community | 481,696 | 40.0% | 42.6% | 40.0% | <.0001 | <.0001 |
| Non-teaching | 173,208 | 14.4% | 7.0% | 14.4% |  |  |
| University | 549,692 | 45.6% | 50.5% | 45.6% |  |  |
|  |  |  |  |  |  |  |
| **Alcohol Use** |  |  |  |  |  |  |
| Not tested | 633,784 | 52.6% | 35.7% | 52.7% | <.0001 | <.0001 |
| No, confirmed by test | 356,342 | 29.6% | 29.2% | 29.6% |  |  |
| Yes, confirmed by test, trace amount | 52,500 | 4.4% | 6.9% | 4.4% |  |  |
| Yes, confirmed by test, beyond legal limit | 161,970 | 13.4% | 28.2% | 13.4% |  |  |
|  |  |  |  |  |  |  |
| **Injury Severity** |  |  |  |  |  |  |
| ISS < 15 | 954,665 | 79.3% | 85.1% | 79.2% | <.0001 | <.0001 |
| ISS >= 15 | 249,931 | 20.7% | 14.9% | 20.8% |  |  |
|  |  |  |  |  |  |  |
| **Injury Type**** |  |  |  |  |  |  |
| Blunt | 1,053,075 | 87.4% | 27.4% | 87.6% | <.0001 | <.0001 |
| Burn | 16,730 | 1.4% | 0.5% | 1.4% |  |  |
| Penetrating | 90,764 | 7.5% | 21.3% | 7.5% |  |  |
| Other/unspecified | 44,027 | 3.7% | 50.8% | 3.5% |  |  |
|  |  |  |  |  |  |  |
| **Injury Location** |  |  |  |  |  |  |
| Home (includes Residential Institution) | 509,063 | 42.3% | 78.4% | 42.2% | <.0001 | <.0001 |
| Other | 695,533 | 57.7% | 21.7% | 57.8% |  |  |
|  |  |  |  |  |  |  |
| **Injury Intent**** |  |  |  |  |  |  |
| Assault | 106,790 | 8.9% | 96.3% | 8.6% | <.0001 | <.0001 |
| Self-inflicted | 20,209 | 1.7% | 0.3% | 1.7% |  |  |
| Unintentional | 1,071,172 | 88.9% | 3.3% | 89.2% |  |  |
| Undetermined/unspecified | 6,425 | 0.5% | 0.1% | 0.5% |  |  |
|  |  |  |  |  |  |  |
| **Injury Mechanism**** |  |  |  |  |  |  |
| Cut/pierce | 47,419 | 3.9% | 16.2% | 3.9% | <.0001 | <.0001 |
| Fall | 513,796 | 42.7% | 2.3% | 42.8% |  |  |
| Fire-burn | 16,730 | 1.4% | 0.5% | 1.4% |  |  |
| Firearm | 43,225 | 3.6% | 5.1% | 3.6% |  |  |
| Struck by, against | 68,087 | 5.7% | 23.2% | 5.6% |  |  |
| Suffocation | 1,164 | 0.1% | 0.4% | 0.1% |  |  |
| Other/unspecified | 514,175 | 42.7% | 52.2% | 42.7% |  |  |
|  |  | 0.0% |  |  |  |  |
| **Mortality** | 39,704 | 3.3% | 1.6% | 3.3% | <.0001 | <.0001 |
|  |  |  |  |  |  |  |
|  |  |  |  |  |  |  |
| *includes white race/missing ethnicity. | |  |  |  |  |  |
| **calculated using primary injury etiology code only. |  |  |  |  |  |  |
| ISS = Injury severity score; Bonferroni = after Bonferroni correction for multiple comparisons |  |  |  |  |  |  |
