# Supplementary material for: Neck and Back Sprain and Hand Flexor Tendon Repair Are More Common in Victims of Domestic Violence Compared With Patients Who Were Not Victims of Domestic Violence: A Comparative Study of 1,204,596 Patients Using the National Trauma Data Bank
Source: J Am Acad Orthop Surg Glob Res Rev. 2021 Sep 2;5(9):e21.00124. doi: 10.5435/JAAOSGlobal-D-21-00124 (PMC8416016; doi:10.5435/JAAOSGlobal-D-21-00124)
Supplement: SUPPLEMENTARY MATERIAL [file jagrr-5-e21.00124-s003.docx]

Supplemental Table 3

| **Supplemental Table 3: Patient, Injury and Hospital Characteristics for Domestic Violence Subgroup, by whether the Abuse Was Perpetrated by an Intimate Partner** | | | | | |  |
| --- | --- | --- | --- | --- | --- | --- |
|  |  |  | **Abuse by parner?** | |  |  |
|  |  |  | **Yes** | **No** | ***P-value (raw)*** | ***P-value (Bonferroni)*** |
|  | **n** | **Percent total** |  |  |  |  |
| All patients | 3191 | 100.0% | 36.6% | 63.4% |  |  |
| n, Age 18-33 | 1310 | 41.1% | 40.7% | 41.3% | <.0001 | <.0001 |
| n, Age 34-48 | 1019 | 31.9% | 35.7% | 29.8% |  |  |
| n, Age 49-69 | 717 | 22.5% | 21.5% | 23.0% |  |  |
| n, Age 70-89 | 145 | 4.5% | 2.1% | 5.9% |  |  |
| Male | 1197 | 37.5% | 19.8% | 47.8% | <.0001 | <.0001 |
| Female | 1994 | 62.5% | 80.2% | 52.3% |  |  |
|  |  |  |  |  |  |  |
| **Race** |  |  |  |  |  |  |
| White, Non-Hispanic | 1300 | 40.7% | 37.0% | 42.9% | <.0001 | <.0001 |
| Black | 971 | 30.4% | 33.5% | 28.7% |  |  |
| Native American | 109 | 3.4% | 2.5% | 4.0% |  |  |
| White, Hispanic | 134 | 4.2% | 2.4% | 5.2% |  |  |
| Other | 399 | 12.5% | 13.5% | 11.9% |  |  |
| Unknown* | 278 | 8.7% | 11.1% | 7.3% |  |  |
|  |  |  |  |  |  |  |
| **Comorbidities** |  |  |  |  |  |  |
| Alcoholism | 545 | 17.1% | 17.5% | 16.9% | 0.66 | 1.0000 |
| Current smoker | 1053 | 33.0% | 31.8% | 33.7% | 0.26 | 1.0000 |
| Diabetes mellitus | 218 | 6.8% | 6.0% | 7.3% | 0.15 | 1.0000 |
| Functionally dependent health status | 18 | 0.6% | 0.4% | 0.6% | 0.44 | 1.0000 |
| Obesity | 191 | 6.0% | 6.2% | 5.9% | 0.75 | 1.0000 |
| Cirrhosis | 20 | 0.6% | 0.7% | 0.6% | 0.75 | 1.0000 |
|  |  |  |  |  |  |  |
| **Primary Payment Method** |  |  |  |  |  |  |
| Medicaid | 806 | 25.3% | 28.0% | 23.7% | 0.0001 | 0.0019 |
| Self Pay | 975 | 30.6% | 26.9% | 32.7% |  |  |
| Private/commercial insurance | 588 | 18.4% | 20.2% | 17.4% |  |  |
| Medicare | 404 | 12.7% | 10.8% | 13.7% |  |  |
| Other | 418 | 13.1% | 14.1% | 12.5% |  |  |
|  |  |  |  |  |  |  |
| **Hospital Teaching Status** |  |  |  |  |  |  |
| Community | 1358 | 42.6% | 35.6% | 46.6% | <.0001 | <.0001 |
| Non-teaching | 223 | 7.0% | 8.1% | 6.4% |  |  |
| University | 1610 | 50.5% | 56.3% | 47.1% |  |  |
|  |  |  |  |  |  |  |
| **Alcohol Use** |  |  |  |  |  |  |
| Not tested | 1139 | 35.7% | 38.9% | 33.9% | 0.0002 | 0.0037 |
| No, confirmed by test | 933 | 29.2% | 30.0% | 28.8% |  |  |
| Yes, confirmed by test, trace amount | 219 | 6.9% | 7.5% | 6.5% |  |  |
| Yes, confirmed by test, beyond legal limit | 900 | 28.2% | 23.7% | 30.8% |  |  |
|  |  |  |  |  |  |  |
| **Injury Severity** |  |  |  |  |  |  |
| < 15 | 2715 | 85.1% | 85.9% | 84.6% | 0.34 | 1.0000 |
| >= 15 | 476 | 14.9% | 14.1% | 15.4% |  |  |
|  |  |  |  |  |  |  |
| **Injury Type**** |  |  |  |  |  |  |
| Blunt | 874 | 27.4% | 21.8% | 30.6% | <.0001 | <.0001 |
| Burn | 16 | 0.5% | 0.8% | 0.4% |  |  |
| Penetrating | 681 | 21.3% | 16.7% | 24.0% |  |  |
| Other/unspecified | 1620 | 50.8% | 60.7% | 45.0% |  |  |
|  |  |  |  |  |  |  |
| **Injury Location** |  |  |  |  |  |  |
| Home (includes Residential Institution) | 2500 | 78.3% | 83.4% | 75.4% | <.0001 | <.0001 |
| Other | 691 | 21.7% | 16.6% | 24.6% |  |  |
|  |  |  |  |  |  |  |
| **Injury Intent**** |  |  |  |  |  |  |
| Assault | 3074 | 96.3% | 96.3% | 96.3% | 0.32 | 1.0000 |
| Self-inflicted | 9 | 0.3% | 0.3% | 0.3% |  |  |
| Unintentional | 106 | 3.3% | 3.3% | 3.4% |  |  |
| Undetermined/unspecified | 2 | 0.1% | 0.2% | 0.0% |  |  |
|  |  |  |  |  |  |  |
| **Injury Mechanism**** |  |  |  |  |  |  |
| Cut/pierce | 518 | 16.2% | 13.7% | 17.7% | <.0001 | <.0001 |
| Fall | 74 | 2.3% | 2.4% | 2.3% |  |  |
| Fire-burn | 16 | 0.5% | 0.8% | 0.4% |  |  |
| Firearm | 163 | 5.1% | 3.0% | 6.3% |  |  |
| Struck by, against | 740 | 23.2% | 16.8% | 26.9% |  |  |
| Suffocation | 14 | 0.4% | 0.8% | 0.3% |  |  |
| Other**/unspecified | 1666 | 52.2% | 62.6% | 46.2% |  |  |
|  |  |  |  |  |  |  |
| **Mortality** | 50 | 1.6% | 1.5% | 1.6% | 0.93 | 1.0000 |
|  |  |  |  |  |  |  |
|  |  |  |  |  |  |  |
| *includes white race/missing ethnicity. |  |  |  |  |  |  |
| **calculated using primary injury etiology code only. | | |  |  |  |  |
| ISS = Injury severity score; Bonferroni = after Bonferroni correction for multiple comparisons | | | | |  |  |
