# Supplementary material for: Neck and Back Sprain and Hand Flexor Tendon Repair Are More Common in Victims of Domestic Violence Compared With Patients Who Were Not Victims of Domestic Violence: A Comparative Study of 1,204,596 Patients Using the National Trauma Data Bank
Source: J Am Acad Orthop Surg Glob Res Rev. 2021 Sep 2;5(9):e21.00124. doi: 10.5435/JAAOSGlobal-D-21-00124 (PMC8416016; doi:10.5435/JAAOSGlobal-D-21-00124)
Supplement: SUPPLEMENTARY MATERIAL [file jagrr-5-e21.00124-s004.docx]

| **Supplemental Table 4: Patients with Included Vertebral Fractures, by Experience of Domestic Violence** | | | | |  |
| --- | --- | --- | --- | --- | --- |
|  |  |  |  |  |  |
|  |  |  | **Experienced domestic violence?** | |  |
|  |  |  | **Yes (% total)** | **No (% total)** | ***P-value**** |
|  | **n** | **% of all patients** |  |  |  |
|  |  |  |  |  |  |
| **All patients with vertebral fractures** | 136,623 |  | 0.08 | 99.92 |  |
|  |  |  |  |  |  |
| n, Age 18-33 | 34,758 | 25.4 | 34.9 | 25.4 | **0.006** |
| n, Age 34-48 | 27,835 | 20.4 | 23.9 | 20.4 |  |
| n, Age 49-69 | 44,681 | 32.7 | 33.9 | 32.7 |  |
| n, Age 70-89 | 29,349 | 21.5 | 7.3 | 21.5 |  |
|  |  |  |  |  |  |
| **Fracture location^+^** |  |  |  |  |  |
| Lumbar (ICD-9 code 805.2)** | 84,186 | 61.6 | 72.5 | 61.6 | 0.06 |
| Thoracic (ICD-9 code 805.4)*** | 71,297 | 52.2 | 37.6 | 52.2 | 0.06 |
|  |  |  |  |  |  |
|  |  |  |  |  |  |
| *P value after correction for multiple comparisons. Values less than 0.05 are considered significant and are presented in bold | | | | | |
| **805.2 = Closed fracture of dorsal [thoracic] vertebra without mention of spinal cord injury) | | | |  |  |
| ***805.4 = Closed fracture of lumbar vertebra without mention of spinal cord injury) | | | |  |  |
| ^+^18,860 patients had both lumbar and thoracic closed vertebral fractures by these criteria | | | |  |  |
| ICD = International classification of disease |  |  |  |  |  |
